# PGSB PlantsDB: updates to the database framework for comparative plant genome research

**DOI:** 10.1093/nar/gkv1130

**Published:** 2015-11-02

**Authors:** Manuel Spannagl, Thomas Nussbaumer, Kai C. Bader, Mihaela M. Martis, Michael Seidel, Karl G. Kugler, Heidrun Gundlach, Klaus F.X. Mayer

**Affiliations:** 1 Plant Genome and Systems Biology, Helmholtz Center Munich – German Research Center for Environmental Health, 85764 Neuherberg, Germany; 2 Division of Computational Systems Biology, Department of Microbiology and Ecosystem Science, University of Vienna, 1090 Vienna, Austria; 3 BILS (Bioinformatics Infrastructure for Life Sciences), Division of Cell Biology, Department of Clinical and Experimental Medicine, Linköping University, SE-558185 Linköping, Sweden

## Abstract

PGSB (Plant Genome and Systems Biology: formerly MIPS) PlantsDB (http://pgsb.helmholtz-muenchen.de/plant/index.jsp) is a database framework for the comparative analysis and visualization of plant genome data. The resource has been updated with new data sets and types as well as specialized tools and interfaces to address user demands for intuitive access to complex plant genome data. In its latest incarnation, we have re-worked both the layout and navigation structure and implemented new keyword search options and a new BLAST sequence search functionality. Actively involved in corresponding sequencing consortia, PlantsDB has dedicated special efforts to the integration and visualization of complex triticeae genome data, especially for barley, wheat and rye. We enhanced CrowsNest, a tool to visualize syntenic relationships between genomes, with data from the wheat sub-genome progenitor *Aegilops tauschii* and added functionality to the PGSB RNASeqExpressionBrowser. GenomeZipper results were integrated for the genomes of barley, rye, wheat and perennial ryegrass and interactive access is granted through PlantsDB interfaces. Data exchange and cross-linking between PlantsDB and other plant genome databases is stimulated by the transPLANT project (http://transplantdb.eu/).

## INTRODUCTION

With the availability of (cost-) efficient next-generation sequencing technologies as well as novel bioinformatic assembly and analysis strategies, plant genome sequencing has entered a new era. Recently, sequence drafts were generated for the large and complex genomes of some of the most economically important cereal crops including barley (1), wheat (2,3) and rye (4). This data significantly promotes our understanding of plant evolution and assists researchers in the much-needed improvement of crops (5). However, due to large genome sizes, high repeat content and polyploidy, genome sequence data generated within these initiatives typically remain unfinished and fragmented for the time being, with heterogeneous and high-volume data sets associated. These challenges strongly ask for dedicated and powerful data integration infrastructures which should also provide intuitive and comprehensive user access. Both data integration and easy access to the data together with the availability of genome sequence data from a variety of related species including models and industrial crops have shown their potential in addressing important biological questions (6). Unfortunately, database solutions and existing interfaces for (finished) plant model genome sequence data often cannot fully account for the storage and representation of these complex genome data, e.g. virtual gene orderings or assemblies of genes on representative model organisms.

Here, we describe the latest updates to the PGSB (Plant Genome and Systems Biology; formerly MIPS) PlantsDB database framework with a special focus on the integration and representation of complex cereal genome data as well as on tools for the (comparative) analysis of plant genomes. Further updates include simplified user navigation and search interfaces/options. PlantsDB is part of the EU-funded transPLANT network which facilitates the exchange, integration and virtual aggregation of plant genome data from distributed resources as well as the development of common standards and protocols.

PGSB PlantsDB can be accessed at http://pgsb.helmholtz-muenchen.de/plant/index.jsp.

### PLANTSDB – new search options and tools

**Figure 1.**
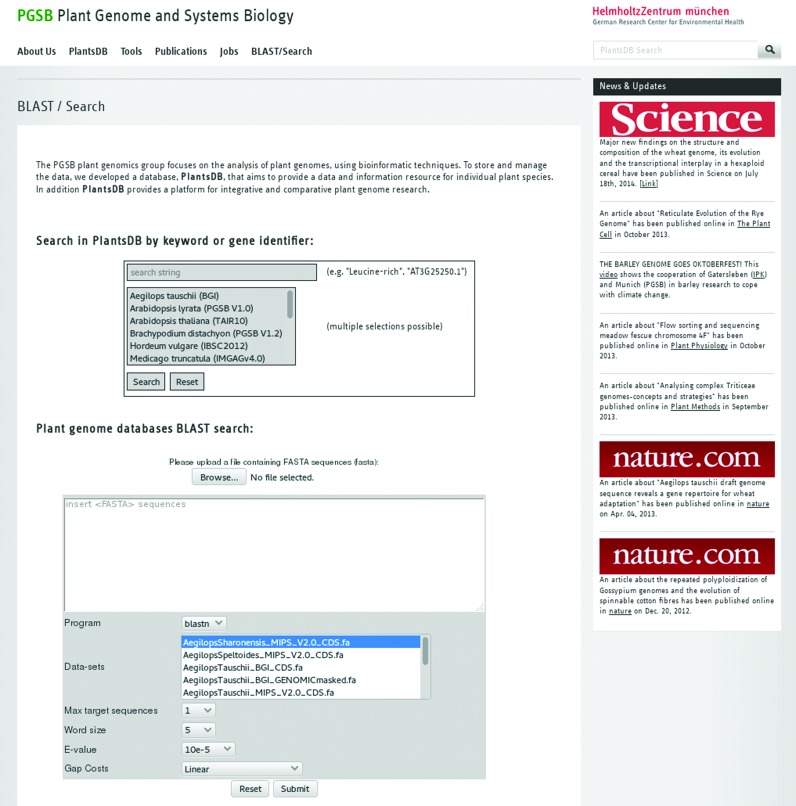
Re-worked PGSB (Plant Genome and Systems Biology; formerly MIPS) PlantsDB search interfaces, providing intuitive keyword searches for gene identifiers, functional gene descriptions and plant genome resources. A new BLAST interface enables sequence similarity searches against genome and CDS/protein sequences from 18 different plant species.

To facilitate intuitive and fast keyword searches on a set of plant gene (function) descriptions, a new search interface was created and linked from the PlantsDB front page (‘PlantsDB Search & BLAST’; Figure 1). For a total of 13 different plant genomes and their corresponding gene predictions, human readable descriptions (‘functional descriptions’) were either derived from curated resources or computed using the AHRD (‘Automatic assignment of human readable descriptions’) tool (6). A filtering option enables searches for specific organisms, combinations of organisms or the entire set. From the results, links to the corresponding and more detailed gene reports, either within PlantsDB or to external reference databases, are provided.

**Figure 2.**
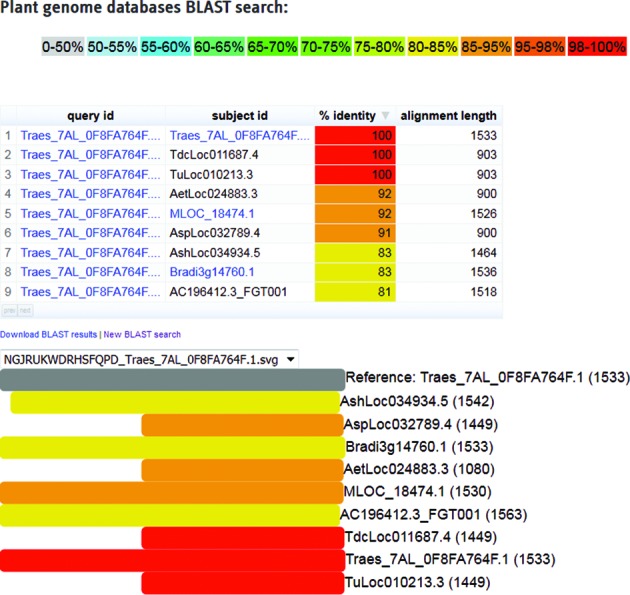
PGSB BLAST search results visualization. Homology matches and alignment visualization of one particular bread wheat gene (Traes_7AL_0F8FA764F.1) and homologous regions to selected progenitor genomes and relatives. The colors depict the sequence identity, whereas the numbers in brackets represent the nucleotide length of the transcripts.

We also implemented a completely new BLAST (7) interface to assist users with their search for homologous sequences across a broad range of plant species. Sequence similarity searches are possible against databases from 18 different species, including wheat (3), several wheat relatives/progenitor genomes (3,8,9), barley (1) and many more crop and model plants. Depending on the input sequence type and species, formatted BLAST databases were made available for coding sequences (CDS), protein sequences and genomic DNA sequence. After a search is completed, hits are visualized in a hierarchical view (Figure 2) and colored with respect to the query sequence identity. Furthermore, links to the corresponding databases with additional information about a specific gene are provided for all hits identified.

Available as a third search component, the transPLANT genome resources registry provides access to a comprehensive, curated and up-to-date collection of plant genome databases and resources. More details on this resource are given in the PlantsDB transPLANT section. Figure 1 shows a screenshot of the updated search functions and the BLAST interface available at PGSB PlantsDB.

In its latest version, both the layout and user navigation of PGSB PlantsDB have been completely re-worked to allow for easier, faster and more intuitive data access.

### PLANTSDB – *triticeae* instances

Many agronomically important crops such as barley, wheat and rye belong to the family of triticeae plants (10,11). The genomes of these crops are typically characterized by their large overall size, high repeat content and complex genetics. With ∼17.1 GB in size the allo-hexaploid genome of bread wheat is more than five times larger than the human genome, exceled also by the genome sizes of barley (∼5.1 GB) and rye (∼8 GB) (12,13). Assembly of these genomes is hampered by an exceptionally high repeat content (up to ∼80% in wheat (3)) on the one hand and, for wheat, by the presence of three highly similar homologous sub-genomes on the other hand.

Nevertheless, draft genome sequences for barley (1), wheat (2,3) and rye (4) were released recently, each generated by dedicated sequencing efforts within international consortia. Novel bioinformatic strategies were used to analyze these genomes and derive the protein-coding gene complements (14). Within these projects, numerous heterogeneous data sets were generated and, in part, integrated with other data from the same or related organisms.

As a consequence, a number of specialized interfaces, viewers and tools were set up within PlantsDB in the past to accommodate novel data types, analysis results, raw data or integrated data sets for cereal genomes (15). This includes interfaces and tools to search, visualize and mine the 5x coverage 454 sequence of the bread wheat genome generated by an UK consortium in 2012 (2,15).

Recently, the International Wheat Genome Sequencing Consortium (IWGSC) released a chromosome-arm sorted whole-genome sequence draft for the bread wheat genome (cultivar Chinese Spring) together with the draft sequences of several wheat progenitors and wheat relatives (such as durum wheat) (3). Gene prediction and functional annotation for all these genomes were performed by PGSB and resulting data is stored within PlantsDB. Online access via PlantsDB standard interfaces and tools to all the IWGSC gene predictions including relatives/progenitor genomes is currently prepared and will be available soon. To assist the cereal research communities, we set up structured and comprehensive FTP download centers for both the barley and the wheat genome (ftp://ftpmips.helmholtz-muenchen.de/plants/barley/public_data/; ftp://ftpmips.helmholtz-muenchen.de/plants/wheat/IWGSC/). Within these, not only gene calls are available for download in several formats but also expression data, whole-genome sequence assemblies, physical and genetic maps plus their integration/anchoring, POPSEQ data (16), GenomeZipper data and repeat annotation.

While these data represent valuable resources for breeders and experimental plant researchers, data interpretation and analysis remains challenging in cereal genomes due to fragmented sequences and the absence of a physical position for a large number of genes. Here, the GenomeZipper concept (17) provides a powerful approach to create linearly ordered, information-rich scaffolds of cereal genomes. GenomeZippers are constructed by making use of gene orders found to be conserved over large portions (‘synteny’) between grass genomes (10,11) and incorporates chromosome sorting, next generation sequencing, array hybridization as well as fl-cDNAs, ESTs and genetic markers. Using this strategy, a total of 49 053 wheat genes could be assigned in the GenomeZipper on corresponding chromosomes recently. GenomeZippers have been constructed for barley (17), bread wheat (3), rye (4) and perennial ryegrass (18) so far and all data including raw sequences have been integrated into PlantsDB along with dedicated visualization interfaces and search options. GenomeZipper data can be queried online by any anchored gene model from one of the grass model organisms (Brachypodium, Sorghum and rice) as well as by any anchored genetic elements such as ESTs, reads or fl-cDNAs. All GenomeZipper results and data are also available for batch download in Excel and/or CSV format from PlantsDB to facilitate in-depth analyses (Barley: http://pgsb.helmholtz-muenchen.de/plant/barley/gz/download/index.jsp; Wheat: ftp://ftpmips.helmholtz-muenchen.de/plants/wheat/IWGSC/genomeZipper/).

### PLANTSDB – comparative genomics and genome analysis tools

#### CrowsNest synteny viewer updates

CrowsNest is an interactive tool to visualize syntenic relationships at macro and micro levels in plant genomes, especially between the genomes of model species and the more complex crop grasses. CrowsNest features a comparative mapping and computation pipeline closely associated with PlantsDB which incorporates genetic, physical and hierarchical (fingerprinted contigs) maps. The tool facilitates the identification and closer investigation of chromosomal rearrangements, inversions and deletions at different resolutions and between two or more chromosomes and/or genomes and therefore promotes the transfer of knowledge between several plant species. CrowsNest views have been computed between genes from the model grasses Brachypodium, Sorghum and rice as well as to the barley genome (15). Lately, we extended the browser with the draft genome from *Aegilops tauschii* (9), which is the putative progenitor of the bread wheat D sub-genome. Because of its reduced genome size and complexity when compared to the allo-hexaploid bread wheat genome, the *Aegilops tauschii* genome serves as good model for its more complex relative. Furthermore, in *Aegilops tauschii* many genes could be anchored on a genetic map (9), providing a robust and comprehensive framework for synteny studies between triticeae genomes and grass model genomes. CrowsNest can be accessed from the ‘PlantsDB Tools’ section as well as from the gene reports of species included in CrowsNest.

#### Accessing gene expression data with the RNASeqExpressionBrowser

**Figure 3.**
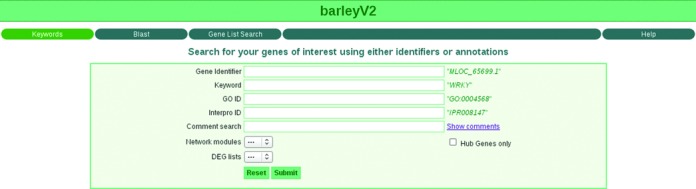
Project entry page of the RNASeqExpressionBrowser with extended search features for gene-to-group mapping (‘network modules’) and for lists of differentially expressed genes.

The RNASeqExpressionBrowser, which is part of PlantsDB (access via ‘PlantsDB Tools’), is a web tool for browsing and visualizing gene expression information (19). It allows searching genes in several ways, such as by gene identifier(s), functional annotations or sequence homology. Here, we present an updated version including a variety of novel features. After quantifying (genome-wide) expression, researchers often make use of clustering strategies for grouping interesting genes, e.g. by hierarchical clustering or supervised machine learning. One of these approaches is network biology, which aims at exploiting the interaction information between pairs of genes and is becoming more and more of a standard approach for analyzing huge data sets. Methods such as weighted correlation network analysis (WGCNA, (20)) allow establishing co-expression networks where groups of closely connected genes, referred to as modules, partition the data into smaller and easier to manageable portions. As the construction of gene-centric networks has become an important component in many studies, the RNASeqExpressionBrowser was extended to support related tasks. In addition to the existing search methods, now also network module membership can be used as a search criterion (Figure 3). Additionally, a filtering for highly connected ‘hub’ genes is possible, as the expression of these genes might be representative for the remaining module members. An alternative approach for narrowing down the list of gene candidates is to study patterns of differential expression. For RNA-seq several tools, e.g. Cufflinks (21) or DESeq (22), have become widely used standards for applying statistics to extract regulated genes. With the latest version of the RNASeqExpressionBrowser it is now also equipped with methods for including and searching information about differential expression.

During expression analysis, annotating or commenting on genes of interest is an important but burdensome task. Therefore, we now included an optional comment feature, which enables adding textual comments to genes. These text entries can then be searched later, e.g. for including references or flagging gene candidates. The entire list of stored comments can also be exported as a PDF.

RNASeqExpressionBrowser is available for download and standalone installation on the client side as well as for two PlantsDB projects using barley expression data (1,23).

### MIPS repeat element database (mips-REdat) and catalog (mips-REcat)

In our previous description of PlantsDB (15) we provided access to the Repeat Element Database (pgsb–REdat) and Repeat Element Catalog (pgsb–REcat) and offered various methods for browsing and downloading repetitive elements. Whereas the pgsb–REdat provides sequences only, especially of importance during the pre-processing of sequences during gene annotation efforts where genomic sequences is normally masked by repetitive elements to prevent false gene calls, the repeat catalog offers a classification of repetitive elements into main groups and, if the order of domains is known, into more specific repeat families. Both resources enable researches to either use the existing classification and sequences for an *in silico*-based detection of repeats or for analyzing more complex and nested repeat insertions.

In order to help researches in automatically detecting and quantifying repeats on their query sequences, we extended the BLAST server with a possibility to also compare query sequences against the 61 730 repeat sequences with known classification based on the latest database version 9.3. It represents an update to the previously reported version 9.0 and includes now additional 11 479 *Gossypium* repeats (24). As outcome of the BLAST search, homology-matching repeats are grouped into repeat families according to the repeat catalog. The alignments between query and repeats might then help to analyze the insertions of various repetitive elements. Access to pgsb–REdat and pgsb–REcat is granted through ‘PlantsDB tools’ or directly at http://pgsb.helmholtz-muenchen.de/plant/recat/index.jsp.

### PLANTSDB – transPLANT

The EU-funded transPLANT (Trans-national Infrastructure for Plant Genomic Science) project is a collaborative effort to enhance data integration, management and analysis of plant genome data and provides services to plant research communities by developing tailored visualization/analysis tools, data exchange protocols and technologies to inter-connect major plant genome resources. A total of 11 different European institutions are organized within several transPLANT workpackages, with PlantsDB being part of the plant genome databases backbone. PGSB is also hosting a plant genome resources registry which is actively maintained and collects and annotates sequence-based databases and resources for both species of agricultural and economic importance and model plants. At date, a total of ∼300 different plant genome resources are registered in the transPLANT registry giving access to associated information such as annotation version, database URL, tools available and data types stored.

Besides regular routine updates and ongoing manual collection and curation of publicly available plant genome database systems, users and database curators can now use an interactive submission and update system to either submit information on new resources or request updates and/or corrections on existing entries.

The registry can be queried both from the PGSB PlantsDB tools section (or directly at http://pgsb.helmholtz-muenchen.de/plant/transplant/genomeResources.jsp) and the official transPLANT web hub at EBI (http://transplantdb.eu/, synchronized with PlantsDB regularly). The transPLANT web hub at EBI also runs an integrated search engine which indexes a number of different PlantsDB services including direct links to CrowsNest views for a given gene identifier and gene reports.

Within transPLANT, a number of user training activities were conceived especially for the analysis and navigation through resources for complex cereal genomes. TransPLANT user training videos describing the use and analysis of distributed barley and wheat genome resources have been produced and are available from PlantsDB (ftp://ftpmips.helmholtz-muenchen.de/plants/user_training/) and http://www.transplantdb.eu/videos.

## CONCLUSION

The PGSB PlantsDB database framework has been significantly enhanced with new tools and interfaces over the last two years and plenty of new data have been integrated into the system, especially for the large and complex genomes of wheat, barley and rye. Close collaboration with user communities and ongoing commitments in several genome sequencing and analysis consortia have guided the development of both novel resources such as the GenomeZipper and facilities for the comparative analysis of data such as CrowsNest and RNASeqExpressionBrowser. We also completely re-worked the database design and the menu structure as well as numerous interfaces and tools such as our BLAST interface to provide a more intuitive and seamless user navigation.

The transPLANT project provides a framework for the integration of heterogeneous plant genome data as well as for data exchange and common services, e.g. integrated searches over multiple databases. We are planning to further utilize transPLANT services in PlantsDB, e.g. by aggregating external genome data from transPLANT partners via web services.
